# Remedies and Their Uses

**Published:** 1906-06-23

**Authors:** 


### Remedies and their Uses.

### Notes on Limitations in the Action of Digitalis.

One of the most curious limitations in the action of digitalis is that it will only produce its characteristic action, of increasing the general vaso-motor tone and of slowing and strengthening the beat of the heart, when the temperature is normal. Thus in an ordinary case of pneumonia or typhoid it is possible to give very large doses of digitalis without producing the least effect on the heart or arterioles. The drug is useless when fever is present. A practical corollary from this is that other medicines must be used in these conditions. As a matter of fact, the only one which will strengthen the cardiac action and increase the vaso-motor tone is strychnine. An excellent rule to adopt is, give ♏ 3 of liq. strychnine hydrochlor. hypodermically every four hours if the pulse-rate is above 120 a minute. We have only seen one case—of pneumonia—where this treatment had to be stopped, owing to slight diarrhœa, or rather too frequent actions of the bowels. The above named amount is not sufficiently large to produce any muscular twitching unless it has to be continued for over forty-eight hours. If this occurs the dose must be diminished. The explanation of this limitation of the action of digitalis is very obscure, but a little light is thrown upon the question by the following facts. In cases of severe attacks of diphtheria, rheumatism, and influenza, the heart is very apt to dilate—sometimes very suddenly. This dilatation may affect either the right or left side of the heart; if the left, it is found that the pulse becomes more feeble and rapid and that the apex of the heart moves outside the nipple-line, and there may be developed a mitral murmur owing to dilatation of the left auriculo-ventricular ring. If the right side of the heart is affected, there is increase of dullness to the right of the sternum and some amount of cyanosis. As is well known, dilation of the heart in these conditions may exceptionally occur with surprising suddenness, and is then usually heralded by vomiting. Now it is found that in these conditions, as in fevers, digitalis will not act—the only drug of any use being, again, strychnine. The treatment is to keep the patient lying absolutely flat and to give strychnine hypodermically. Unless the cardiac tissue is damaged by some previous disease or chronic degeneration this procedure is generally successful. As the heart recovers the strychnine may gradually be left off, but the patient should be kept lying flat until the cardiac action has slowed down to within ten or fifteen beats per minute of its normal rate. A somewhat surprising phenomenon in these cases, especially in diphtheritic cardiac failure, is that the rapid feeble cardiac action continues for very many weeks, perhaps eight or ten, or even more, and then rather suddenly, within a week or so, becomes normal. Repair is long delayed and then occurs rapidly. The fact that digitalis does very little if any good in such cases indicates that it does not act on degenerated cardiac muscle fibre even when the temperature is normal. This probably explains why digitalis will not act on the rapid feeble heart of fevers; the rapidity and feebleness are not due simply to the temperature, but to the action on the cardiac muscle fibres of the toxins which are also producing the temperature.

### On Caton's Blistering Treatment for Rheumatic Endocarditis.

Caton, of Liverpool, introduced some years ago a method of treating rheumatic endocarditis by means of blisters. It is as follows: The patient is kept strictly in bed, and blisters of the size of a shilling are put or painted on the precordium in a circle round the apex—one by one. A blister is put say an inch or so to the left of the apex, allowed to rise, snipped, and a dressing applied; a couple of days later another is applied a little below and to the right of the first, and so on. The results claimed for this treatment are that many cases of mitral endocarditis recover and lose all trace of a murmur. The fact—recovery in many cases—may be granted; but it is probably not a result of the blisters, but due to the patient's being kept in bed. Blisters may act in two ways, either reflexly or by altering the condition of the blood. As no nerves enter any of the cardiac valves, a series of blisters on the precordium cannot act reflexly on endocarditis; and there is no special virtue in applying them in a ring round the apex. The case is quite different from that of applying them over a chronically inflamed joint which can be acted on reflexly. Further, there is no evidence that blisters act, either for good or evil, on the general toxæmia of rheumatism. Another explanation, then, should be forthcoming of the good results of Caton's treatment. It has been found that the rheumatic virus has a marked action on the cardiac muscle as well as, in some cases, on the cardiac valves. The cardiac muscle is very apt to undergo an acute degeneration, which weakens its action and impairs its tone. Owing to this the heart dilates, the cardiac dullness extends to the left, and the apex moves out. At the same time its action becomes feebler. If the apex moves out more than half an inch outside the nipple-line a murmur may be developed at the apex—due to regurgitation into the left auricle through a widened mitral orifice, without there being any endocarditis present in the mitral valve. As the heart gradually recovers the apex moves in, the murmur disappears, the heart's action becomes slower and stronger. A mitral murmur developing in the course of rheumatic fever may thus be due either to endocarditis or to dilatation of the heart; the former condition probably always persists, the latter is recovered from. The success of Caton's treatment lies in the fact that the patients are kept lying flat in bed; this gives the best possible condition for rheumatic dilatation to be recovered from. The fact that a larger proportion of cases lose their murmurs if they are kept lying down than when they are soon got up appears to indicate that exertion, if undertaken before cardiac dilatation is recovered from, is very apt to render it permanent.

